# Role of the oxygen supply position in the circuit during continuous positive airway pressure therapy: a randomized cross-over simulation study

**DOI:** 10.1007/s11325-026-03589-z

**Published:** 2026-02-07

**Authors:** Zoe Bousraou, Lara Benning, Christina Spengler, Silvia Ulrich, Martin Lüchinger, Esther Irene Schwarz

**Affiliations:** 1 Department of Pulmonology, Sleep Disorders Centre and Ventilation Unit, University Hospital Zurich, Zurich, Switzerland; 2 University of Zurich, Zurich, Switzerland; 3 Exercise Physiology Lab, Institute of Human Movement Sciences and Sport, ETH Zurich, Zurich, Switzerland; 4 Department of Pulmonology Head of the Sleep Disorders Centre and Ventilation Unit, University Hospital Zurich, Zurich, Switzerland

**Keywords:** Oxygen therapy, Continuous positive airway pressure, Lung simulator, Sleep-disordered breathing

## Abstract

**Purpose:**

Continuous Positive Airway Pressure (CPAP) is the main treatment for patients with obstructive sleep apnoea (OSA). Hypoxemic patients with sleep-disordered breathing may require CPAP and additional low-flow oxygen. However, specific recommendations on where to attach the oxygen bleed-in connector are lacking.

**Methods:**

This randomised cross-over trial assessed the fraction of inspired oxygen (FIO₂) in a simulated OSA patient’s lung resulting from different rates of low-flow oxygen (1, 2 and 3 l/min) added via three different bleed-in connector locations under different levels of either fixed pressure (5, 9 and 12 cmH_2_O) or constant flow (5, 10 and 15 l/min) (repeated 3x in random order). The three locations tested in a single-limb circuit with a vented full-face mask were: (1) near the CPAP mask between the mask and tubing, (2) at the tube outlet of the device, and (3) directly connected to the oxygen-port of the device.

**Results:**

The bleed-in connector positioned near the tube outlet generally produced the highest and near the CPAP mask the lowest FiO₂. At the highest pressure and flow, FiO₂ remained more stable across the locations and oxygen flows of 1–3 l/min, compared to lower pressure and lower flow. FiO₂ differences between locations were notably more pronounced under different flows, ranging from 0 to 22% relative change, compared to a more limited range of 0 to 8.3% under varying CPAP pressures.

**Conclusion:**

We recommend positioning the oxygen bleed-in connector at the device tube outlet for CPAP devices without an oxygen port, particularly when using lower pressures.

## Introduction

Obstructive sleep apnoea (OSA) is characterised by intermittent hypoxaemia due to obstructive apnoeas and/or hypopnoeas during sleep. OSA-associated intermittent hypoxaemia can usually be corrected by successful treatment of OSA. Intermittent hypoxaemia is the key pathophysiological driver of adverse vascular effects of OSA [1]. Patients with more severe OSA-specific intermittent hypoxaemia as assessed e.g. by the hypoxic burden [2], have a higher risk of adverse cardiovascular outcome. Continuous positive airway pressure (CPAP) is the treatment of choice for OSA, mechanically splinting the upper airway with positive pressure and thus preventing repetitive collapse and the pathophysiological consequences of obstructive events such as intermittent hypoxaemia [3, 4]. However, many patients with OSA have combined forms of sleep-disordered breathing, in particular a combination of OSA and hypoxaemia. In addition to OSA-specific intermittent hypoxaemia, hypoxemic patients have a low baseline oxygen saturation due to impaired gas exchange. This is particularly common in patients with cardio-pulmonary comorbidities such as chronic obstructive pulmonary disease (COPD), obesity-hypoventilation syndrome (OHS), interstitial lung disease (ILD) and pulmonary arterial hypertension (PAH) [5, 6]. Consecutively, a combination of CPAP and oxygen is required to correct the sleep-disordered breathing in these hypoxemic patients [7–9]. The addition of oxygen to CPAP is recommended for patients with an indication for long-term oxygen therapy and OSA, e.g. in COPD-OSA-overlap syndrome [9]. However, guidelines on long-term oxygen supplementation or positive airway pressure therapy lack specific recommendations on how to provide and titrate supplemental oxygen therapy via CPAP or other positive airway pressure therapy devices [10–16]. The oxygen requirement is higher during sleep than when awake and increases with higher flow of the positive airway pressure device, as the oxygen is diluted with more room air [17]. CPAP devices used for OSA treatment typically lack an oxygen port. Supplemental oxygen is usually supplied via an oxygen adapter that is connected to the CPAP circuit. The bleed-in oxygen connector can theoretically be fitted between the mask and the tube or at the tube outlet of the CPAP device. Reliable FiO_2_ delivery however depends on effective CPAP therapy and lack of large unintentional leaks. Efficient application of supplemental oxygen via CPAP in hypoxemic patients with OSA is particularly important to prevent pulmonary hypertension and right heart failure. Oxygen alone is not recommended in hypoxemic patients with symptomatic OSA as it does not prevent upper airway collapse or address the pathophysiological consequences of OSA and may prolong apnoeas and hypopnoeas [18] The question that arises is whether changing the position of the low-flow oxygen-input in the circuit as well as varying pressure and flow conditions of the positive airway pressure therapy device influences how much oxygen reaches the lungs. We hypothesised that the supply of oxygen via bleed-in connector at the CPAP tube outlet would result in a higher fraction of inspired oxygen than via a bleed-in connector between the vented mask and the tubing near the intentional leak.

## Materials and methods

### Study design and lung simulator

**Fig. 1:**
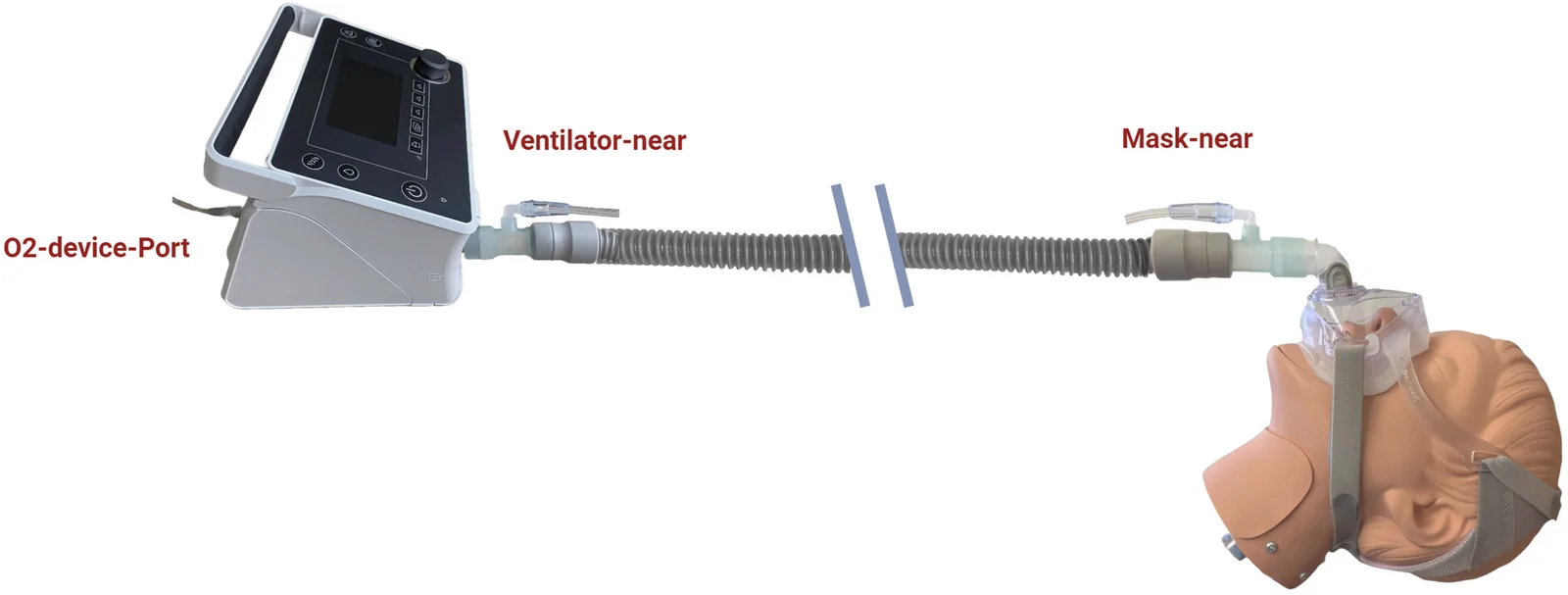
The three bleed-in oxygen connector locations in the circuit

In a randomised cross-over study, fraction of inspired oxygen (FiO_2_) with 1, 2, and 3 l/min of supplemental oxygen delivered via three different locations in a positive airway pressure device circuit (single tube and vented mask) was assessed in a lung simulator (Dual Adult Lung Simulator, Michigan Instruments, Grand Rapids, MI, USA) during 5, 9 and 12 cmH_2_O of CPAP and during 5, 10 and 15 l/min of constant airflow (Prisma VENT50-C, Löwenstein Medical, Bad Ems, Germany) in random order. The pressures tested represent a typical CPAP pressure range in OSA. CPAP devices and home ventilators generally use single-limbs with vented masks and, alongside intentional leakage to avoid CO_2_ rebreathing, unintentional leaks are common. Devices react to compensate leaks by increasing the flow, which can dilute the supplemented oxygen. Consecutively, the FiO_2_ achieved with oxygen supply via different positions in the circuit was measured not only at different constant pressures, but also at different fixed flow rates. The three locations for oxygen supply were a bleed-in connector positioned either between the CPAP mask and the tube (mask-near) or between the tube and the tube outlet of the positive airway pressure device (ventilator-near) and the oxygen port of the ventilator (Fig. 1).

## Set-up and assessments

A lung simulator (Dual Adult Lung Simulator, Michigan Instruments, Grand Rapids, MI, USA) was used to simulate an OSA patient with a healthy lung through use of compliance and resistance settings. An adult head simulator with an open mouth and nose was attached to the lung simulator to allow placement of a CPAP mask and use of a positive airway pressure device (Fig. 1). An airway resistor of Rp20 cmH_2_O/l/s and a lung compliance of 0.05 l/cmH_2_O were used as recommended by Michigan Instruments. The left lung was termed the “driving lung”, representing the body’s respiratory muscle pump. The right lung termed “breathing lung” was attached to the left “lifting lung” via a lung-coupling clip, to allow the simulation of spontaneous passive breathing. The “breathing lung” was the focus of this study, representing an OSA patient’s lung under spontaneous breathing conditions. A breathing hose connected the Michigan Instruments MII Breathing Simulation Module (BSM) to a Gas Tank filled with room air, generating an estimated maximum pressure of 45 cmH2O in the driving lung. This setup enabled rhythmic breathing and spontaneous lung simulation of the right lung via a coupling chip. The BSM was set to produce 12–14 breaths per minute (remained constant at 13 breaths per minute), with an inspiratory time of 1.5 s. The BSM conditions remained consistent for all trials. The lung compliance of the left lung was set to 0.10 l/cmH_2_O, a physiologic compliance as recommended by Michigan Instruments.

The Prisma VENT50-C device (Löwenstein Medical, Bad Ems, Germany) was used for CPAP and constant flow generation, respectively. The hose connected to the device was attached to a ResMed AirFit F10 XS mask (ResMed, SanDiego, California), which was placed on the dummy head, connected to the breathing lung. CPAP pressure or flow were adjusted based on the respective trial condition (5, 9 or 12 cmH_2_O and 5, 10 or 15 l/min). The oxygen input connector was placed either mask-near or ventilator-near based on the condition of the trial being tested. Low flow oxygen via oxygen tank to oxygen connector was added (1, 2 or 3 l/min). The lung simulator fed information into the PneuView System software program which was run on a laptop connected via USB. Prior to data collection, the PneuView references were set to Lung View: Right Lung (the “breathing lung” being tested), Expected Breath Rate: 0-100 bpm, Gas Reference Conditions: ATPX (Ambient Temperature and Pressure, Relative (X) humidity (Actual Conditions)), Barometric Pressure: 760 mmHg, Resistance: Rp20 and Compliance: 0.05.

Baseline FiO2 was 21%. Through the FiO_2_ sensor located on the simulated “breathing lung”, FiO_2_ values were continuously recorded for each condition for each of the three oxygen port locations mentioned above. An algorithm in Excel was used to randomise the test runs (54 conditions repeated 3 times) and a minimum of 2 min were taken between each trial to allow the oxygen to completely leave the simulated lung. Each condition was performed for 2 min (prior testing trials showed that the FiO_2_ stabilised after 1 min for all conditions). FiO_2_ returned to baseline 21% after one minute.

## Tested locations and conditions

18 conditions (3 oxygen flow rates (1, 2 and 3 l/min) in 3 pressure settings (5, 9, 12 cmH_2_O) and at 3 device flow rates (5, 10, 15 l/min)) were tested in all three oxygen supply locations (mask-near, ventilator-near, built-in oxygen port, Fig. 1) three times each in random order (162 measurements).

## Statistics

Calculations and visualisations were conducted in R Studio to evaluate changes in FiO_2_ measurements under the various conditions. The absolute FiO_2_ difference and relative percent change between each of the three bleed-in connector locations was calculated for each CPAP and flow condition. Separate plots were generated to illustrate FiO₂ differences between conditions, with CPAP pressure and flow analysed independently to ensure clarity in interpretation. The mean of the three repeated measurements per condition was used for comparisons between the three different locations.

## Results

### Repeatability of FiO2

There was a very high repeatability for FiO₂ measurements across all three test runs with a consistent FiO_2_ and a coefficient of variation of 0% for all 54 tested conditions (Table 1).

**Table 1:** Three repeated measurements of FiO_2_ with 1, 2 and 3 l/min O_2_ supply via the three different locations (mask-near, ventilator-near, oxygen Port of the ventilator) for the three different constant pressures and the three different constant flow rates

	Run 1	Run 2	Run 3	Run 1	Run 2	Run 3	Run 1	Run 2	Run 3
	5 cmH2O	5 cmH2O	5 cmH2O	9 cmH2O	9 cmH2O	9 cmH2O	12 cmH2O	12 cmH2O	12 cmH2O
1 l/min O2mask-near	23	23	23	23	23	23	22	22	23
1 l/min O2 ventilator-near	23	23	23	23	23	23	23	23	23
1 l/min O2 ventilator-port	24	24	23	23	23	23	23	23	23
2 l/min O2 mask-near	24	24	24	24	24	23	24	24	24
2 l/min O2 ventilator-near	25	25	25	25	24	24	24	24	24
2 l/min O2 ventilator-port	26	26	25	25	24	25	24	24	24
3 l/min O2 mask-near	26	25	26	25	25	25	25	26	26
3 l/min O2 ventilator-near	27	27	27	26	26	26	26	26	25
3 l/min O2 ventilator-port	28	28	28	25	26	26	26	26	25
	5 l/min	5 l/min	5 l/min	10 l/min	10 l/min	10 l/min	15 l/min	15 l/min	15 l/min
1 l/min O2 mask-near	24	24	24	24	24	24	24	24	24
1 l/min O2 ventilator-near	27	26	27	26	26	26	24	24	24
1 l/min O2 ventilator-port	25	25	25	25	25	25	24	24	24
2 l/min O2 mask-near	27	27	27	26	27	26	27	27	27
2 l/min O2 ventilator-near	31	31	31	30	30	31	27	27	27
2 l/min O2 ventilator-port	28	28	28	28	28	28	27	27	27
3 l/min O2 mask-near	30	29	30	30	30	29	29	29	30
3 l/min O2 ventilator-near	37	36	36	34	34	34	29	29	29
3 l/min O2 ventilator-port	32	32	32	31	31	32	30	30	30

## Differences in FiO2 between the positions of the oxygen Inlet at different CPAP pressures

**Fig. 2:**
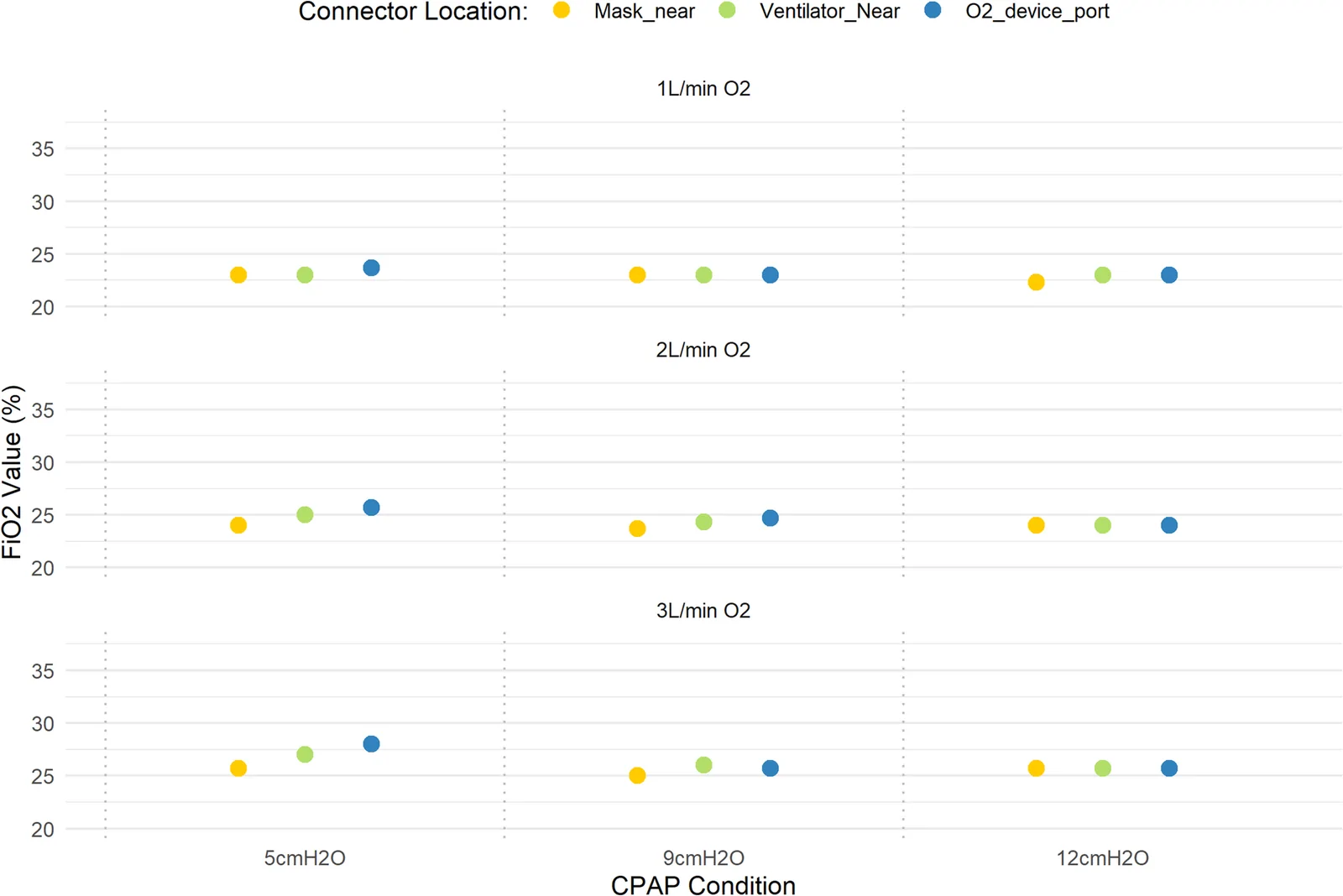
FiO₂ values for each bleed-in connector location at three CPAP pressures (5, 9 and 12 cmH_2_O) under low-flow oxygen of 1,2 and 3 l/min O_2_

Overall, the lowest FiO_2_ values resulted when oxygen was supplied via the mask-near bleed-in connector (Fig. 2; Tables 1 and 2). FiO2 was generally higher when oxygen was supplied via the ventilator-near connector compared to the mask-near connector (Fig. 2; Tables 1 and 2). At lower CPAP pressure however, the highest FiO2 was reached via oxygen port of the ventilator. The relative percent change between the three bleed-in oxygen connectors for the three CPAP pressures 5, 9 and 12 cmH_2_O ranged from 0 to 9% as shown in Table 2. The absolute difference in percent FiO₂ that occurred between bleed-in connectors ranged from 0 to 2.3 FiO₂. The localisation of the oxygen supply was no longer relevant for FiO_2_ with the combination of higher CPAP pressure (12 cmH_2_O) and at least 2 l/min O_2_.

**Table 2:** Absolute difference in FiO_2_ and the relative percent change for each CPAP pressure (5, 9 and 12 cmH_2_O) between the three locations (oxygen port, ventilator-near, mask-near) under 1, 2 and 3 l/min O_2_

Connector Location Comparison	O_2_ (L/min)	CPAP condition	Difference in FiO_2_ (%)	Relative Percent Change (%)
Ventilator-near – Mask-near	1	5 cmH2O	0	0
O2-device-port – Ventilator-near	1	5 cmH2O	0	0
O2-device-port – Mask-near	1	5 cmH2O	0.7	3.0
Ventilator-near – Mask-near	1	9 cmH2O	0	0
O2-device-port – Ventilator-near	1	9 cmH2O	0	0
O2-device-port – Mask-near	1	9 cmH2O	0	0
Ventilator-near – Mask-near	1	12 cmH2O	0.7	3.1
O2-device-port – Ventilator-near	1	12 cmH2O	0	0
O2-device-port – Mask-near	1	12 cmH2O	0.7	3.1
Ventilator-near – Mask-near	2	5 cmH2O	1.0	4.2
O2-device-port – Ventilator-near	2	5 cmH2O	0.7	2.8
O2-device-port – Mask-near	2	5 cmH2O	1.7	7.1
Ventilator-near – Mask-near	2	9 cmH2O	0.6	2.5
O2-device-port – Ventilator-near	2	9 cmH2O	0.4	1.7
O2-device-port – Mask-near	2	9 cmH2O	1.0	4.2
Ventilator-near – Mask-near	2	12 cmH2O	0	0
O2-device-port – Ventilator-near	2	12 cmH2O	0	0
O2-device-port – Mask-near	2	12 cmH2O	0	0
Ventilator-near – Mask-near	3	5 cmH2O	1.3	5.1
O2-device-port – Ventilator-near	3	5 cmH2O	1.0	3.7
O2-device-port – Mask-near	3	5 cmH2O	2.3	9.0
Ventilator-near – Mask-near	3	9 cmH2O	1.0	4.0
O2-device-port – Ventilator-near	3	9 cmH2O	-0.3	-1.2
O2-device-port – Mask-near	3	9 cmH2O	0.7	2.8
Ventilator-near – Mask-near	3	12 cmH2O	0	0
O2-device-port – Ventilator-near	3	12 cmH2O	0	0
O2-device-port – Mask-near	3	12cmH2O	0	0

Role of CPAP pressure: Overall, FiO_2_ was higher at lower CPAP pressure for a given oxygen flow rate. At a pressure of 12 cmH₂O, relative changes in FiO₂ between locations were small (0-3.1%) (Table 2). In contrast, lower pressures of 5 cmH₂O and 9 cmH₂O introduced slightly larger FiO₂ differences between locations (up to 9 and 4.2% relative change, respectively).

Role of oxygen flow rate: A higher oxygen flow rate resulted in larger variations of FiO₂ values between locations. An oxygen input of 1 l/min resulted in a FiO₂ variation of 0-3.1%, 2 l/min had a range of 0-7.1% relative percent change and 3 l/min had a range of 0–9% change between locations (Table 2). Maximal absolute differences in FiO_2_ between 1, 2 and 3 l/min were reached at low CPAP pressure of 5 cmH_2_O (max. 4.3%).

In summary, the mask-near connector appears to have slightly lower FiO_2_ values in most conditions compared to the other two locations that are closer to the ventilator, when comparing under same CPAP pressure and oxygen flow rate conditions.

## Differences in FiO2 between the position of the oxygen connection at different ventilator flow rates

**Fig. 3:**
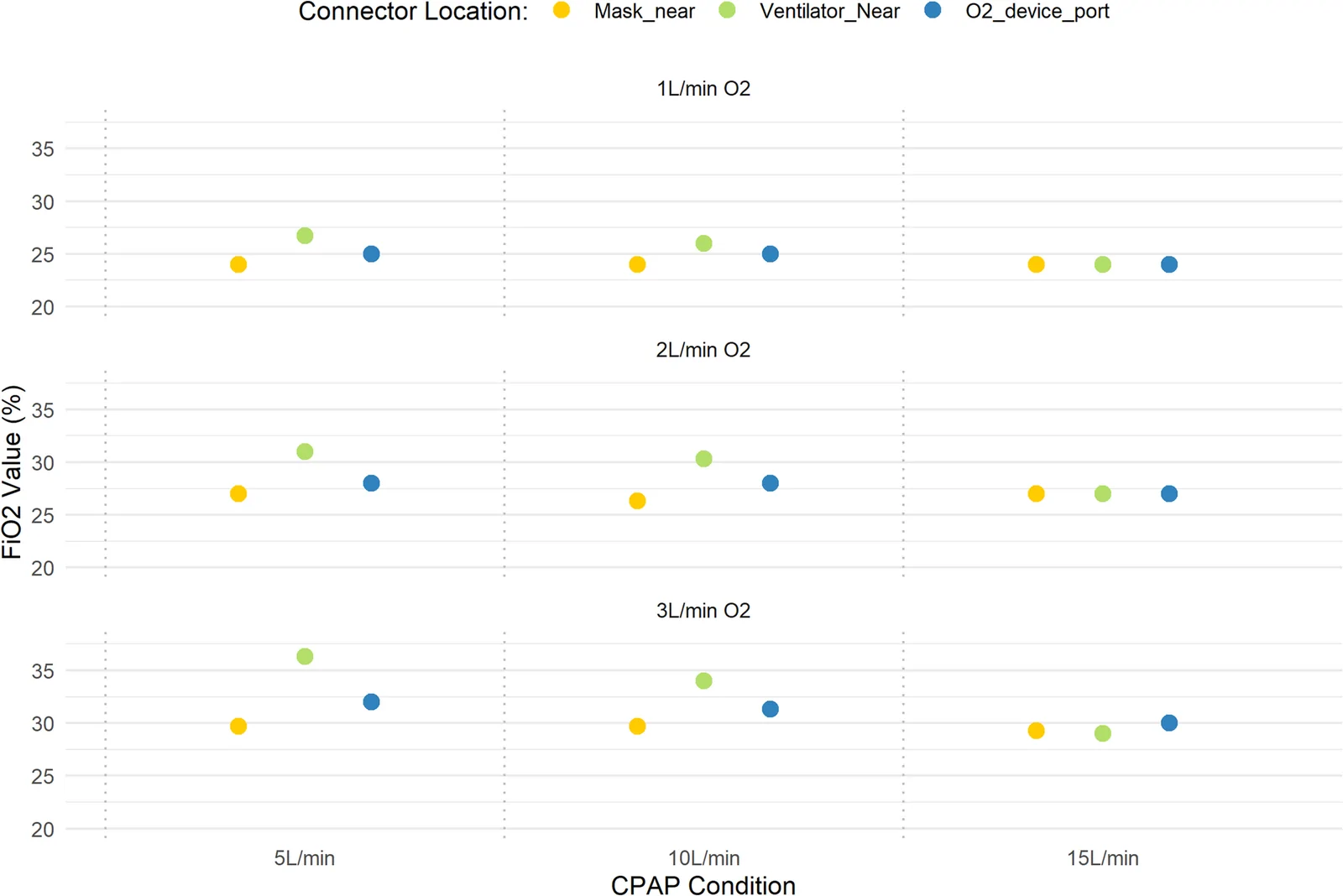
FiO_2_ values for each bleed-in connector location at three ventilator flow rates (5, 10 and 15 l/min) under low-flow oxygen rates of 1, 2 and 3 l/min O_2_)

The difference in FiO_2_ between the three oxygen supply positions differed much more for a given constant ventilator flow rate (0-6.6% absolute change and 0–22% relative change) compared to the fixed pressures. The highest FiO_2_ was reached by oxygen supplementation via the ventilator-near bleed-in connector (Figure 3 and Tables 1 and 3). The mask-near bleed-in connector consistently resulted in the lowest FiO_2_ value. However, the difference became less relevant at the highest flow rate of 15 l/min (similar to the highest pressure of 12 cmH2O).

**Table 3:** The absolute difference in FiO_2_ and the relative percent change for each ventilator flow rate (5, 10 and 15 l/min) between the three locations under 1, 2 and 3 l/min O_2_

Connector Location Comparison	O2 (L/min)	CPAP condition	Difference in FiO_2_ (%)	Relative Percent Change (%)
Ventilator-near – Mask-near	1	5 l/min	2.7	11.3
O2-device-port – Ventilator-near	1	5 l/min	-1.7	-6.4
O2-device-port – Mask-near	1	5 l/min	1.0	4.2
Ventilator-near – Mask-near	1	10 l/min	2.0	8.3
O2-device-port – Ventilator-near	1	10 l/min	-1.0	-3.9
O2-device-port – Mask-near	1	10 l/min	-1.0	-4.0
Ventilator-near – Mask-near	1	15 l/min	0	0
O2-device-port – Ventilator-near	1	15 l/min	0	0
O2-device-port – Mask-near	1	15 l/min	0	0
Ventilator-near – Mask-near	2	5 l/min	4.0	14.8
O2-device-port – Ventilator-near	2	5 l/min	-3.0	-9.7
O2-device-port – Mask-near	2	5 l/min	1.0	3.7
Ventilator-near – Mask-near	2	10 l/min	4.0	15.2
O2-device-port – Ventilator-near	2	10 l/min	-2.3	-7.6
O2-device-port – Mask-near	2	10 l/min	1.7	6.5
Ventilator-near – Mask-near	2	15 l/min	0	0
O2-device-port – Ventilator-near	2	15 l/min	0	0
O2-device-port – Mask-near	2	15 l/min	0	0
Ventilator-near – Mask-near	3	5 l/min	6.6	22.2
O2-device-port – Ventilator-near	3	5 l/min	-4.3	-11.9
O2-device-port – Mask-near	3	5 l/min	2.3	7.7
Ventilator-near – Mask-near	3	10 l/min	4.3	14.5
O2-device-port – Ventilator-near	3	10 l/min	-2.7	-7.9
O2-device-port – Mask-near	3	10 l/min	1.6	5.4
Ventilator-near – Mask-near	3	15 l/min	-0.3	-1.0
O2-device-port – Ventilator-near	3	15 l/min	1.0	3.5
O2-device-port – Mask-near	3	15 l/min	0.7	2.4

Role of ventilator flow rate: When the ventilator flow was set to 15 l/min, FiO₂ differences between connector locations and low-flow oxygen inputs (1–3 l/min) remained minimal (0-3.4% relative difference). However, at lower fixed flows of 5 l/min and 10 l/min, FiO₂ variations between locations were more substantial (0-22.2% and 0-15.2% relative change respectively) between locations as shown in Table 3; Fig. 3.

Role of oxygen flow rate: Higher oxygen flow rates resulted in larger differences in FiO₂ values between locations. An oxygen input of 1, 2 and 3 l/min, resulted in a relative FiO₂ variation between locations of maximal 11.3%, 15.2% and 22.2%, respectively (Table 3).

**Fig. 4:**
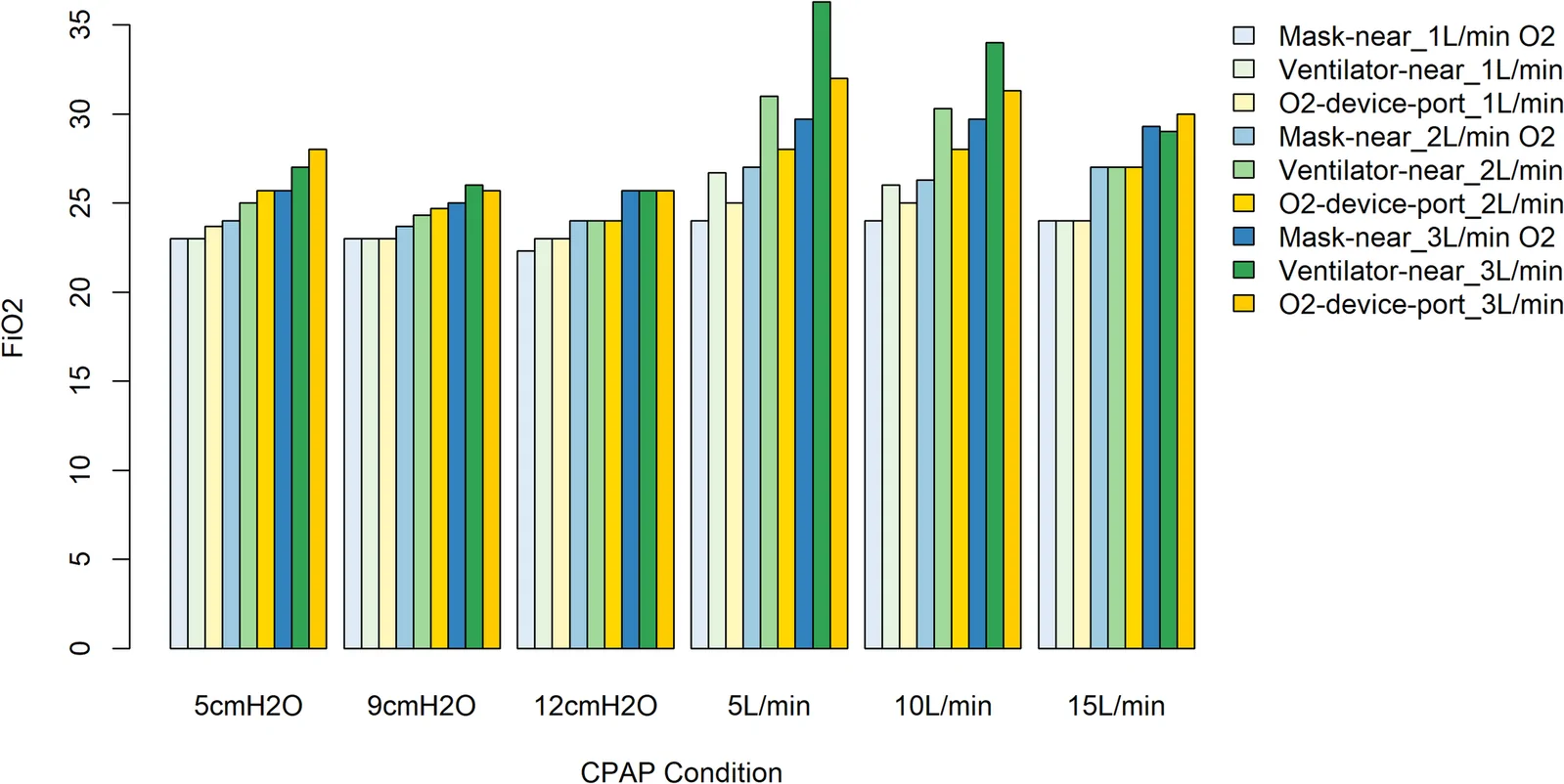
Bar plot of average FiO_2_ (%) values for all test runs. Each colour represents a O_2_-bleed-in connector location, different shades of the colour represent different oxygen flow rates

To summarise, the highest FiO2 was achieved at fixed ventilator flow rates of up to 10 l/min and different oxygen flow rates when oxygen was fed in via the bleed-in connector close to the ventilator (Fig. 4). The FiO2 was slightly higher than when applied via the oxygen port of the ventilator and significantly higher than when applied via the connector close to the mask. At high flow rates of 15 bl/min, localisation no longer played such a significant role.

## Discussion

In this randomised cross-over study, the relevance of the localisation of low-flow oxygen supplementation in the circuit of a positive airway pressure therapy device for the home environment was investigated. The study examined how changing the location of the bleed-in oxygen connector of a CPAP circuit affected the FiO₂ levels in a simulated lung, representing a healthy adult with OSA under low flow-oxygen and typical CPAP pressures and flows (also comparable to minute ventilation as seen in home mechanical ventilation). Supplementation of 1–4 l/min oxygen via a CPAP device is a common scenario in hypoxemic patients with OSA, e.g. COPD, OHS, ILD or PAH. The oxygen concentration administered to the patient via CPAP will depend on the machine flow and the supplemental oxygen flow [17, 19]. Higher pressure provided by higher flow as well as mask leakage dilute oxygen flow [17]. A FiO_2_ requirement of 25–30% is common. Oxygen guidelines lack specific recommendations for nocturnal or long-term oxygen supplementation with positive airway pressure devices. However, not only the device flow and the oxygen flow but also the position of the bleed-in connector influences the amount of oxygen reaching the lungs. In contrast to some home mechanical ventilators, CPAP devices usually lack an oxygen port. Therefore, a bleed-in oxygen connector must be installed in the circuit, either close to the ventilator or close to the mask. This study has shown that the highest FiO_2_ can generally be achieved via the ventilator-near bleed-in connector (between ventilator tube outlet and tube) - with the exception of a low CPAP pressure of 5 cmH2O, at which the FiO_2_ was slightly higher when oxygen was supplied via the ventilator oxygen port. The results indicate that the bleed-in connector positioned near the CPAP mask overall produced the lowest FiO₂ values in each scenario. This finding is in line with a study testing oxygen supply via a side port of the mask and via a port near the bilevel positive airway pressure ventilator showing a consistently lower FiO_2_ when oxygen was placed though side port in the mask.

Higher oxygen flow rates resulted in larger variation of FiO_2_ between bleed-in oxygen connector locations. At a higher CPAP pressure of 12 H_2_O and a higher fixed ventilator flow of 15 l/min, the differences in FiO_2_ between the oxygen input localisations became negligible. In order to achieve a higher FiO_2_, the feed at the ventilator tube outlet is therefore particularly important in the case of lower CPAP pressures and when higher oxygen flow rates are required. One explanation for the lower FiO2 in case of mask-near oxygen supply is the proximity to the intentional leakage. Intentional leaks are needed during CPAP therapy and are a result of the vent holes in the mask which allow for carbon dioxide elimination [20]. When oxygen is added closer to the CPAP mask, there is a higher likelihood of oxygen loss through the mask’s ventilation holes, as the oxygen bleed-in connector is located directly adjacent to the intentional leak ports of the mask. The air and oxygen may not have had enough time to mix within the circuit and oxygen is lost via ventilation holes in the mask. Conversely, introducing oxygen at the start of the tubing near the CPAP device allows for better mixing, resulting in more consistent oxygen delivery to the mask and lungs resulting in higher FiO_2_ [21].

As the oxygen demand during sleep is higher due to the lower minute ventilation and the increase in ventilation-perfusion mismatch, and the correction of nocturnal hypoxaemia is important to avoid pulmonary hypertension, ensuring adequate oxygen delivery via a CPAP device is very important in hypoxemic patients with OSA with combined sustained and intermittent hypoxaemia.

The study has some limitations. In a real-world setting, FiO_2_ could be affected by changes in mask leakage, which affects flow and thus oxygen dilution, by sleep stage, which affects minute ventilation, and by sleep position, which affects minute ventilation and ventilation-perfusion mismatch. The pathophysiology of the respiratory muscle pump, lung and gas exchange of OSA patients with comorbidities such as COPD, OHS, ILD or PAH are not reflected. As a simulation study, the setup cannot fully replicate patient physiology, including variable leak patterns, mucosal humidity, dynamic airway resistance, and ventilation-diffusion-mismatch, and the differences in FiO₂ observed in the simulator may not translate linearly to arterial oxygenation in humans. The absolute FiO_2_ values achieved here under the various pressure and flow conditions can therefore not be directly transferred to patients with cardiopulmonary comorbidities. However, the constant and controlled conditions of the lung simulator, here with normal compliance, allow a reliable comparison of the oxygen supply localisations. In follow-up studies, the role of the oxygen supply position can be investigated using the lung simulator with altered compliance, in humans with OHS or COPD, and also under bilevel positive pressure ventilation. Validation in human subjects will be an essential next step for future research. To increase the real-world applicability of these findings, a future study should investigate the effects of varying degrees of mask leak. Another limitation is that the FiO_2_ sensor only provided whole numbers. Incorporating a sensor that reports an extra decimal point may improve accuracy and allow detection of smaller variations. The reliability of FiO_2_ measurements seen between repeated test runs suggests that the measurement process and setup are reliable and minimally influenced by random errors or external factors. Consequently, any observed differences between conditions are more likely to reflect actual effects rather than measurement variability, increasing the robustness of the conclusions drawn from the data. An advantage compared to the only other study we found comparing the sites of oxygen delivery during positive airway pressure in a circuit for home positive airway pressure therapy is that FiO2 was not measured in the mask in our study, but more reliably in the lungs [19].

## Conclusions

This study emphasizes the importance of bleed-in oxygen connector placement and avoidance of unintentional leakage to avoid flow increases for optimising oxygen supplementation via CPAP therapy in hypoxemic patients with OSA. We recommend positioning the oxygen bleed-in connector at the device tube outlet for CPAP devices without an oxygen port. This is particularly important when lower pressures are used and when a higher oxygen flow is required. The recommendation to position the connection close to the device - not the mask - should be included in future oxygen and CPAP guidelines.

## Data Availability

Data are available from the corresponding author upon a reasonable request.
